# Retroperitoneal abscess complicated with necrotizing fasciitis of the thigh in a patient with sigmoid colon cancer

**DOI:** 10.1186/1477-7819-7-74

**Published:** 2009-10-07

**Authors:** Yuji Takakura, Satoshi Ikeda, Masanori Yoshimitsu, Takao Hinoi, Daisuke Sumitani, Haruka Takeda, Yasuo Kawaguchi, Manabu Shimomura, Masakazu Tokunaga, Masazumi Okajima, Hideki Ohdan

**Affiliations:** 1 Department of Surgery, Division of Frontier Medical Science, Programs for Biomedical Research, Graduate School of Biomedical Science, Hiroshima University, 1-2-3 Kasumi, Minami-ku, Hiroshima, 734-8551, Japan; 2 Department of Endoscopic Surgery and Surgical Science, Graduate School of Biomedical Science, Hiroshima University, Hiroshima, Japan

## Abstract

**Background:**

Necrotizing fasciitis of the thigh due to the colon cancer, especially during chemotherepy, has not been previously reported.

**Case presentation:**

A 67-year-old man admitted to the hospital was diagnosed with sigmoid colon cancer that had spread to the left psoas muscle. Multiple hepatic metastases were also found, and combination chemotherapy with irinotecan and S-1 was administered. Four months after the initiation of chemotherapy, the patient developed gait disturbance and high fever and was therefore admitted to the emergency department of our hospital. Blood examination revealed generalized inflammation with a high C-reactive protein level. Computed tomography of the abdomen and pelvis showed gas and fluid collection in the retroperitoneum adjacent to the sigmoid colon cancer. The abscess was locally drained under computed tomographic guidance; however, the infection continued to spread and necrotizing fasciitis developed. Consequently, emergent debridement was performed. The patient recovered well, and the primary tumor was resected after remission of the local inflammation.

**Conclusion:**

Necrotizing fasciitis of the thigh due to the spread of sigmoid colon cancer is unusual, but this fatal complication should be considered during chemotherapy for patients with unresectable colorectal cancer.

## Background

Necrotizing fasciitis (NF) is a rare and life-threatening soft-tissue infection. Aggressive surgical management is required in the early stage in order to reduce the associated high mortality rate, which ranges from 20% to 40%[1]; however, it is often difficult to diagnose NF in the early stages.

NF is usually caused not only by trauma to the skin, such as that induced by insect bites, scratches, and abrasion, but also by surgical wounds in the perineum and lower extremities[2]. Other less common causes include perforated or penetrated diverticulitis, ruptured appendix, and inflammatory bowel diseases[3]. To date, few reports of NF caused by colon cancer have been published. We present a rare case of NF of the thigh during chemotherapy due to the retroperitoneal spread of sigmoid colon cancer.

## Case Presentation

**Figure 1:**
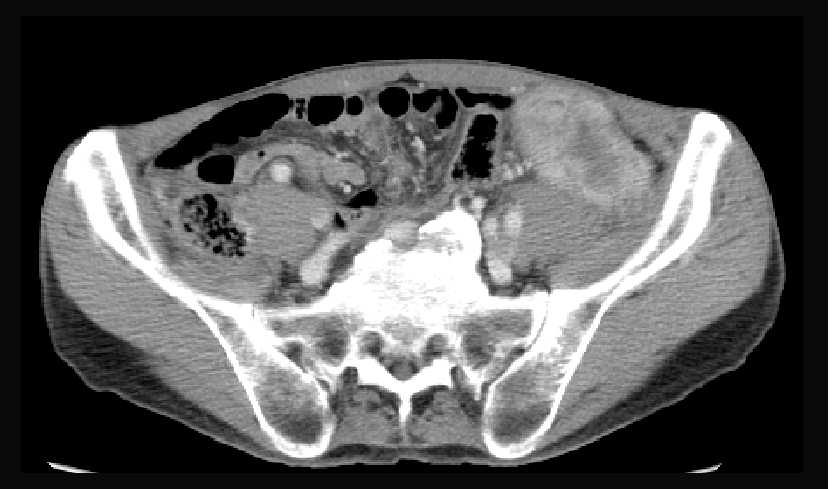
**Sigmoid colon cancer invading to the retroperitoneum at the time of initial diagnosis**.

A 67-year-old man, who was healthy earlier, was referred to our hospital for a month-long history anorexia. On the basis of the results of a computed tomography (CT) scan and gastrointestinal endoscopy, the patient was diagnosed with unresectable sigmoid colon cancer that had spread to the retroperitoneum (Figure 1); multiple liver metastases were also detected. Subsequently, combination chemotherapy with S-1 and irinotecan was administered.

**Figure 2:**
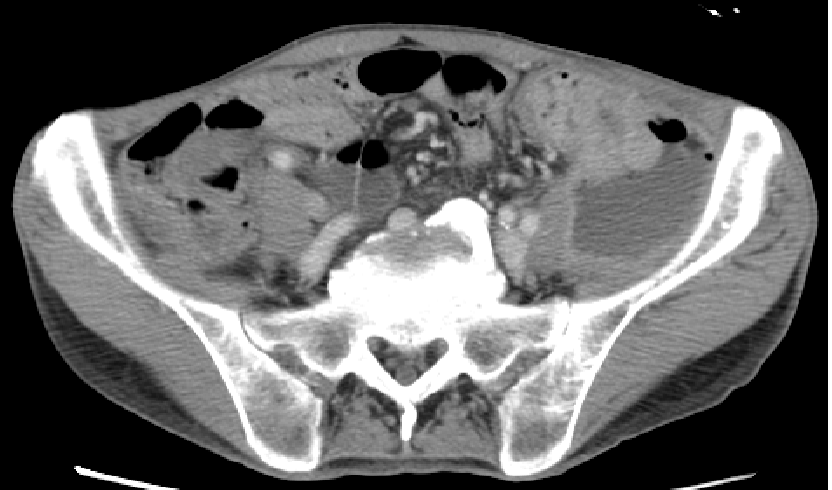
**Retroperitoneal abscess adjacent to the sigmoid colon tumor**.

**Figure 3:**
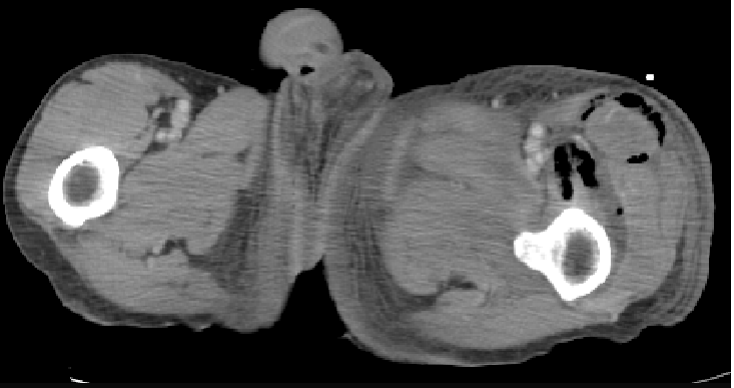
**Abnormal air accumulation in the subcutaneous space of the left thigh**.

Four months after the initiation of chemotherapy, he was readmitted to the hospital for dyskinesia of the left lower extremity and high fever. Blood examination data indicated leukopenia (white blood cell count, 2500 cells/μL), and a high C-reactive protein (CRP) level (16.7 mg/dL). A CT scan showed fluid and gas collection in the retroperitoneum adjacent to the primary tumor (Figure 2). This condition was diagnosed as a retroperitoneal abscess and emergent CT guided drainage of the abscess was performed. A pigtail catheter was inserted into the abscess and pus with gas and odor was drained; an infection caused by gas-producing anaerobic microorganisms was strongly suspected. The patient recovered temporarily, but high fever, crepitus, and diffuse swelling in the left thigh appeared 4 days after the drainage. A CT scan of the pelvis and lower extremity revealed a fluid and gas tracking from the retroperitoneum into the intramuscular plane of the grossly enlarged left thigh (Figure 3), although the size of the abscess had drastically reduced as a result of the drainage. A presumptive diagnosis of necrotizing fasciitis of the left thigh was made, and the patient was immediately taken to the operation room. A wide debridement of the external fascia was performed to reveal the healthy tissue, the retroperitoneum was drained again, and loop ileostomy was created. The patient was admitted to the intensive care unit and administered intravenous antibiotics (carbapenem). Microbiological culture of the pus revealed the presence of *Escherichia coli *and other anaerobic bacteria. The patient showed good postoperative recovery, and the primary tumor was resected 2 months after the first surgery. The operative findings indicated that the cancerous lesion and the tissues surrounding it were firmly attached to the left retroperitoneum. Multiple liver and peritoneal metastases were also detected. Palliative resection of the primary tumor was performed in order to prevent the recurrence of retroperitoneal inflammation. On the basis of the operative findings, the tumor was classified as a T4 (invading the psoas muscle), N1, and M1 (liver and peritoneum), and the patient was clinically diagnosed with stage IV cancer according to the definitions laid down by the International Union Against Cancer (UICC). The patient was given oxaliplatinm 5-fluorouracil, and folinic acid (modified FOLFOX6) therapy, but, he died due to cancer 8 months after the second surgery.

## Discussion

NF is a serious soft-tissue infection that causes secondary necrosis of the subcutaneous tissues. It can occur in any region of the body but most commonly occurs in the abdominal wall, extremities, and perineum.

It has been reported that NF has a high morbidity and mortality rate because of its acute and rapidly progressive course. The outcome of NF is rendered poor most importantly by delays in its diagnosis and surgical debridement. Thus, early diagnosis of necrotizing soft-tissue infections followed by administration of intravenous antibiotics and surgical intervention is the best way of decreasing the mortality associated with this aggressive infection. Clinical features of NF include high fever with chills, tenderness over the affected area along with changes in skin color, and palpable crepitus[1].

It is well known that perineal NF, termed as "Fournier's gangrene," is caused by rectal cancer or periproctal abscess[4], and there are several reports on NF due to colorectal cancer involving the abdominal wall[5,6]. However, NF of the thigh due to the spread of colorectal cancer, as observed in the present case, is extremely rare. Literature review reveals only 3 such cases [7-9]. Colon cancer usually spreads intraperitoneally, and its spread in the retroperitoneal direction is relatively rare.

In the 3 previously reported cases, symptoms of NF preceded the diagnosis of colorectal cancer; thus, to our knowledge, this is the first reported case in which NF developed during chemotherapy for the treatment of colorectal cancer.

In the present case, we inserted only the pig tail catheter immediately after the diagnosis of retroperitoneal abscess, because we thought that the patient may not tolerate the stress of radical surgery. However, we realized that this was a wrong strategy because NF developed eventually and additional debridement was required. Fortunately, the patient showed good postoperative recovery, however, we believe that NF, a serious complication, could have been avoided if the radical treatment had been initiated earlier.

Recent advances in chemotherapy for colorectal cancer (e.g., cytotoxic agents such as irinotecan, oxaliplatin, and the fluoropyrimidines, and bevacizumab and cetuximab) have improved the median survival period of patients with unresectable colorectal cancer [10-15]. Patients with unresectable colorectal metastases who were treated with the latest multidrug systemic therapy have shown a median period of 18-20 months[13,15].

Therefore, chemotherapy is currently the first line of treatment for patients with unresectable colorectal cancer. Palliative resection of the primary lesion is rarely performed when there are no symptoms of primary cancer, such as intestinal obstruction or bleeding.

Although there are several reports have stated that primary tumor resection contributes to prolonged survival in patients with incurable colorectal cancer[16,17], there is no consensus on the same among medical oncologists and surgeons [18-20].

Specifically, a high incidence of bowel perforation and delayed wound healing have been observed in patients treated with bevacizumab[21]. Therefore, adequate care should be taken to prevent perforation and penetration following NF in such patients. In addition, NF might indicate a serious complication, and result in high mortality.

Our reported case highlights the importance of the removal of the primary tumor in an aymptomatic patient as an attempt to avoid concomitant serious complications.

Retroperitoneal abscess and NF are rare complications of colorectal cancers that can potentially be fatal, particularly in patients who are immunocompromised because of chemotherapy. In the presence of these unclear risk factors, accurate and rapid clinical judgment and a careful consideration of balance between the risks and benefits are necessary before performing a palliative surgery.

## Conclusion

Colon cancer could be a cause of unexpected retroperitoneal abscess followed by NF of the thigh, and NF should be considered during the diagnosis of colon cancer. Early diagnosis and treatment can help reduce the mortality rate associated with NF.

## Abbreviations

NF: necrotizing fasciitis; CT: computed tomography; CRP: C-reactive protein.

## Competing interests

The authors declare that they have no competing interests.

## Authors' contributions

YT participated in treatment of the patient, collected case details, literature search and draft the manuscript. SI participated in treatment of the patient and helped to draft the manuscript. MY, TH, DS, HT, YK, MS and MT participated in treatment of the patients. MO and HO participated in treatment planning of the patient and helped to draft the manuscript. All authors read and approved the final manuscript.

## Consent

Written informed consent was obtained from the patient for publication of this case report and any accompanying images. A copy of the written consent is available for review by the Editor-in-Chief of this journal.
